# An optimization method for measuring the stomata in cassava (*Manihot esculent*a Crantz) under multiple abiotic stresses

**DOI:** 10.1515/biol-2022-0993

**Published:** 2024-11-11

**Authors:** Muqing Ma, Jinbao Gu, Zhen-Yu Wang

**Affiliations:** Hainan Key Laboratory for Sustainable Utilization of Tropical Bioresource, College of Tropical Crops, Hainan University, Haikou, 570228, Hainan, China; Institute of Nanfan & Seed Industry, Guangdong Academy of Sciences, No. 10 Middle Jianghai Avenue, Haizhu District, Guangzhou, Guangdong, 510316, China

**Keywords:** cassava (*Manihot esculenta* Crantz), stomata, rhodamine 6G, abiotic stress

## Abstract

As a gateway for gas exchange, pores regulate the transport of air and water in carbon assimilation, respiration, and transpiration to quickly adapt to environmental changes. Therefore, the study of stomatal movement characteristics of plants is helpful to strengthen the understanding of the mechanism of plant response to multi-environmental stress, and can improve the function of plant resistance to stresses. The stomatal movement of *Arabidopsis* leaves was observed by staining the stomata with rhodamine 6G, but this method has not been reported in other plant leaf stomata studies. Taking cassava as an example, the correlation between cassava stomatal movement and cassava response to stress was observed by using and improving the staining method. Rhodamine 6G is a biological stain widely used in cell biology and molecular biology. It was found that 1 μM rhodamine 6G could stain the stomata of cassava without affecting stomatal movement (*n* = 109, *p* < 0.05). In addition, we proposed that stomata fixed with 4% concentration of formaldehyde after staining were closest to the stomatal morphology of cassava epidermis, so as to observe stomatal movement under different environmental stresses more accurately. Previous methods of measuring stomatal pore size by autofluorescence of cell wall needs to fix the cells for 6 h, but Rhodamine staining can only be observed in 2 min, which greatly improves the experimental efficiency. Compared with the traditional exfoliation method (e.g., *Arabidopsis*), this method can reduce the damage of the leaves and observe the stomata of the whole leaves more completely, so that the experimental results are more complete. In addition, the method enables continuous leaf processing and observation. Using this method, we further compared four different cassava varieties (i.e., KU50, SC16, SC8, and SC205) and found that there are differences in stomatal density (SD) among cassava varieties, and the difference in the SD directly affects the stress resistance of cassava (*n* = 107, *p* < 0.001). This finding has important implications for studying the mechanism of plant response to environmental stress through stomata.

## 1 Introduction

Cassava (*Manihot esculenta* Crantz) is a vegetatively propagated tuber crop native to Brazil and later spread to South America, Africa, and Asia covering more than 100 countries, and is now the main food crop for about 800 million people worldwide due to its year-round properties [1–3]. Especially in tropical and subtropical regions, it is favored because of its ability to withstand a variety of adverse environmental conditions [4]. Cassava storage roots are rich in starch and are also used as raw materials in the food processing industry [5]. In addition to being a major ingredient for food consumption, cassava is also used in the manufacture of medicines, livestock feed, and biofuels [6]. But drought, salinization, low temperature, and other environmental stresses pose a threat to the improvement of cassava yield and quality, so it is an important research topic to study the drought tolerance mechanism of cassava [7–10]. Cassava adaptation is enormously complicated in response to these environmental stresses. So far, drought-related genes and proteins have been identified [11–14]. In addition, low soil moisture conditions lead to the close of stomata for survival [15]. Necessarily, drought stress closes the stomata which result in lower net photosynthetic rate and transpiration rate of cassava leaves, and then inhibits the plant growth and development [16].

Stomata exchange 95% of gas between the leaf and the atmosphere [17]. Stomata are formed from two guard cells. The guard cells are usually surrounded by accessory cells that are morphologically different from their epidermal neighbors and are the main pathways for water loss and carbon dioxide uptake [18,19]. Controlling transpiration by reducing stomatal pore size can improve drought resistance of plants [20]. Photosynthesis, on the other hand, requires carbon dioxide to enter the inner mesophyll cells through the stomata. Larger pore sizes facilitate this process, but also lead to elevated levels of transpiration [21].

Stomata emergence is an important landmark in evolution of land plants and their adaptation to dry stress [22]. However, stomata have also become an entry point for pathogens. Stomatal movement is regulated by turgor pressure generated by ion and water channel proteins on the plasma membranes of guard cells [23]. Stomatal pore size is tightly regulated by environmental stimuli, including water deficit, pathogen attack, light fluctuations, and CO_2_ levels [19,24,25]. Furthermore, endogenous factors control the movement of stomata, such as abscisic acid (ABA), Ca^2+^, and reactive oxygen species (ROS) levels under various conditions [23–28]. Therefore, the research on stomata will help develop stress-resistant varieties of cassava.

Numerous studies have shown that many plant species alter stomatal development by modulating stomatal size (SS) and stomatal density (SD) in response to long-term changes in environmental stimuli [29]. However, studies on stomatal movement in cassava leaves have not been reported. To fill the gap in cassava stomata research, we revealed that cassava stomata can be stained by rhodamine 6G and examined the response process of cassava stomata to light, abdominic acid, drought, and salt stress. Furthermore, we explored whether there was a correlation between the differences in SD among the four cassava varieties and the response mechanism of cassava to abiotic stress. In conclusion, we have developed a simple, rapid, and non-damaging method for measuring cassava stomata that will be of great value for investigating the mechanisms underlying the response of cassava stomata to biotic and abiotic stresses. Similarly, it is important to develop new stress-resistant cassava lines to mitigate the impacts of global warming and climate change on agriculture.

## 2 Materials and methods

### 2.1 Plant material and growth conditions

Cassava cultivar, KU50, SC16, SC8, and SC205, were tissue cultured in the Murashige and Skoog (MS) culture medium (MS salts, 2% sucrose, 2 μM CuSO_4_, 0.3% phytagel, pH 5.8) (Sigma-Aldrich Crop Ltd, MO, USA) [30]. Twenty-day-old seedlings were transplanted into grey plastic pots (four seedlings per pot) with bottom holes. An air pump was used to maintain normal ventilation. Cassava culture seedlings were grown under long day conditions (16 h-light/8 h-dark, photosynthetically active radiation of 100–120 μmol m^−2^ s^−1^) at 28°C and 50% relative humidity.

### 2.2 Solutions and treatment

To examine the effect of different signals on stomatal movement, leaves were detached from plants and floated with the abaxial side turned down in a petri dish containing MES/KOH buffer (5 mM KCl, 10 mM MES, 50 μM CaCl_2_, pH 6.15) (Tiangen Co., Ltd, Beijing, China). For the treatments, 50 μM ABA (Tiangen Co., Ltd, Beijing, China), 150 mM NaCl (Beyotime Co., Ltd, Shanghai, China), or 300 mM sorbitol (Tiangen Co., Ltd, Beijing, China) in MES/KOH buffer were used. Unless stated otherwise, all treatments were performed under above grown conditions.

For stomatal staining, rhodamine 6G (Sigma-Aldrich Crop Ltd, MO, USA) at final concentration of 1 μM (dissolved in water) was used. It was freshly prepared from 1 mM stock solution (in water), which was kept in dark. During staining, formaldehyde (Aladdin Co., Ltd, Shanghai, China) was added to staining solution.

### 2.3 Selection of formaldehyde concentration

Twelve cassava seedlings with similar growth conditions were selected. A leaf of appropriate size was selected from each seedling and stripped so that the lower epidermis of the leaf was downward. Then, 12 leaves at a similar developmental stage were fully expanded and floated in a petri dish containing MES/KOH buffer to maintain equilibrium and stomatal opening. Three technical replicates were performed to ensure the accuracy of the experiment. After 2 h of continuous light incubation in a constant light chamber with 25 incandescent bulbs as the light source, 50 μM ABA (Tiangen Co., Ltd, Beijing, China) was added and the leaves were imaged at 0, 1, and 2 h [31,32].

For cell staining, we prepared staining solutions of different concentrations. Formaldehyde at 2, 4, 6, and 8% were added in the staining solution. Cassava leaves were dipped into the staining solutions with different concentrations of formaldehyde in 1.5 mL centrifuge tubes for 2 min and then imaged.

### 2.4 Influence of rhodamine 6G staining on stomatal movement

#### 2.4.1 Comparison of rhodamine 6G staining and epidermis peels

Six cassava leaves were prepared at similar developmental stage and put in petri dishes containing MES/KOH buffer. Petri dishes were maintained under light at 25°C for 2 h to fully open the stomata. Then leaves were treated with 50 μM ABA for 1 and 2 h. One leaf half was stained in the staining solution and then examined under LEICA DM5000 B microscope. Another epidermis peels of leaf half were prepared, then immediately analyzed under microscope.

#### 2.4.2 Comparison of pre-stained and post-stained

Six fully expanded leaves were prepared at a comparable developmental stage, and put in petri dishes containing MES/KOH buffer. Cassava leaves were placed in MES/KOH buffer for 2 h for equilibration and stomata opening. Then leaves were treated as follows: one part of the cassava leaves was stained in the formaldehyde-free solution, and then it was treated with 50 μM ABA (pre-stained), whereas the other part was treated with 50 μM ABA directly without pre-staining, then stained immediately before microscopic analysis (post-stained). Leaves were imaged at 0, 1, and 2 h.

#### 2.4.3 Response to light and dark after rhodamine 6G staining

Twelve cassava leaves (three leaves per treatment) were prepared and put in petri dish containing MES/KOH buffer. Petri dishes were placed either under light or in dark for 2 h. Then one part of the cassava leaves was stained in staining solution containing 4% formaldehyde for 2 min and returned to MES/KOH buffer. Another part was stained in the formaldehyde-free solution. Images were taken and returned to the corresponding petri dishes. Cassava leaves previously maintained under light were transferred into dark, whereas those from dark were transferred into light conditions for 30 min. The leaves were then imaged after treatment.

### 2.5 Image processing and stomatal aperture (SA) analyzing

**Figure 1:**
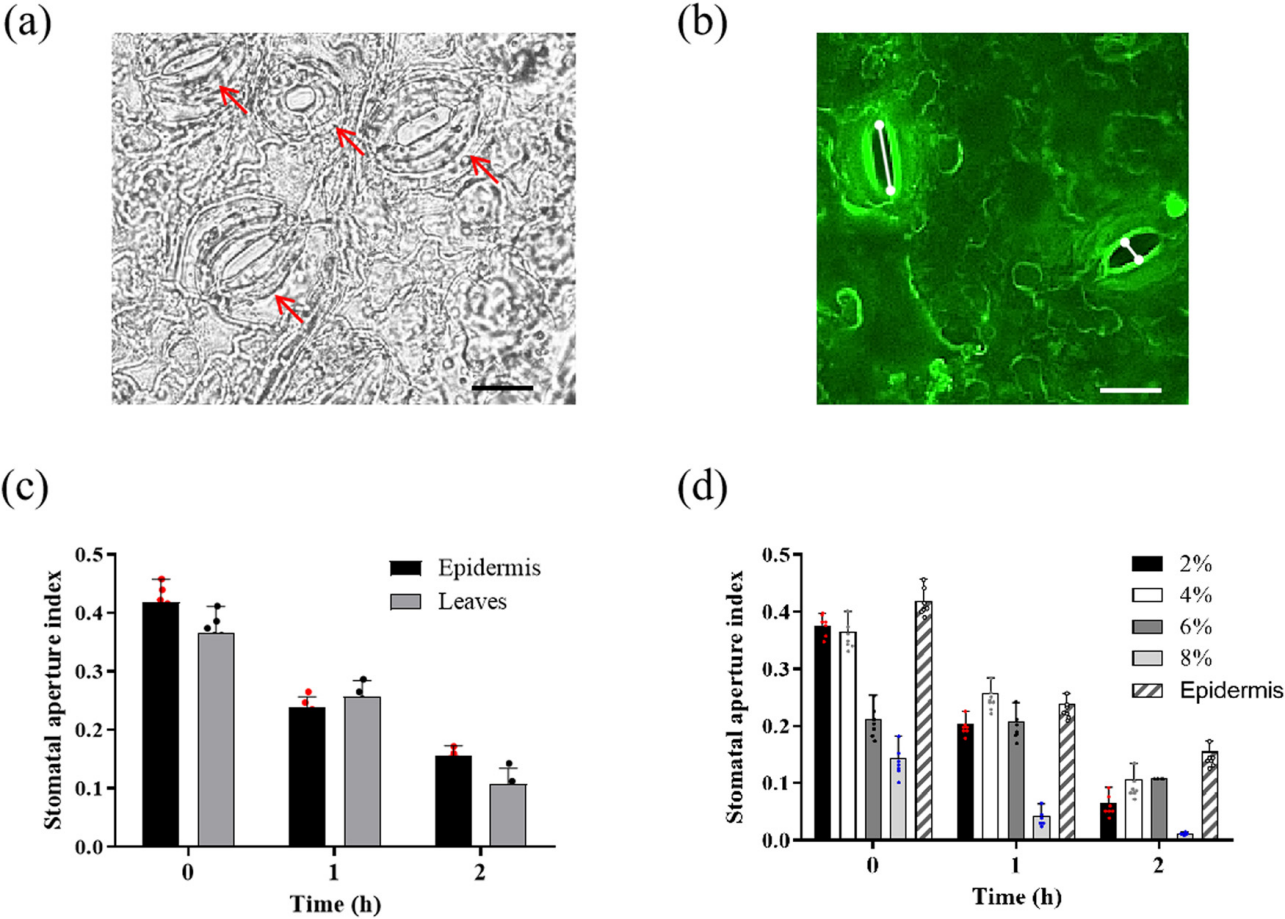
Type of cassava stomata and the condition of rhodamine 6G staining. (a) Stomatal shape of cassava leaf observed under LEICA DM5000 B microscope was kidney shape. (b) One-month old cassava leaves were pre-incubated in MES/KOH buffer under light conditions for 2 h and then stained with 1 μM rhodamine 6G for 2 min. White lanes were the width and the length of stomata. (c) After treatment with 50 μM ABA for 0, 1, and 2 h, the SAs were compared between epidermis and intact leaves. (d) After treatment with 50 μM ABA for 0, 1, and 2 h, the SAs were compared between epidermis and leaves fixed by different concentrations of formaldehyde (2, 4, 6, and 8%). Bar = 20 μm.

The stained abaxial part of cassava leaves are placed on a slide. H_2_O was added to the slide, and the slide was carefully covered with the cover glass (not to leave bubbles). Surrounding water was absorbed with paper, and then the images of at least five different views of each sample were taken by the microscope. SAs were measured using ImageJ software. The width and the length of the SAs were measured as shown in Figure 1b. SA was calculated by division of aperture width through the length. At least 60 stomata that were selected per leaf were measured, and three leaves per treatment/time point were used for statistical analysis. Images were captured at following settings: 20× objective, green fluorescence protein filter, using LEICA application suite V4.2 software. Data were analyzed by a two-tailed paired Student’s *t*-test.

### 2.6 Quantitative real-time PCR (qRT-PCR) analyses

Thirty-day-old seedlings grown on MS medium were immersed in 70 mL sorbitol solution (300 mM), ABA solution (50 μM), NaCl solution (150 mM), and dehydration directly (0, 1, 2, and 4 h) to simulate drought or salt stress on leaves. RNA was extracted from treated cassava leaves by Eastep^®^ Super total RNA extraction kit (Promega, China), and used for reverse transcription through the EasyScript^®^ One-Step gDNA Removal and cDNA Synthesis Super Mix kit (TRANSGEN Tech Ltd, China). qRT-PCR was performed using the TransStart Tip Green qPCR SuperMix (Transgene, Co. Ltd, China) and a Roche LightCycle^®^ 96 System. MeUBQ was used as an internal control and the primers are listed in Table S1. The final volume of the reaction is 20 µL and the qRT-PCR condition was used as follows: denatured at 94°C for 30 s; 45 cycles of 94°C for 5 s, 60°C for 15 s and 72°C for 10 s, with melting and cooling.

## 3 Result

### 3.1 Cassava has a kidney shaped stomata

Stomata were a specialized epidermal structure in land plants, consisting of guard cells. The stomatal complex is a functional unit composed of guard cells and their surrounding accessory cells [17]. The expansion and contraction of guard cells can regulate SA and then regulate gas exchange and transpiration [33]. Guard cells can be characterized as dumbbell-shaped or kidney-shaped [17]. In order to study the type of cassava stomata, we peeled off lower epidermis of SC8 and examined it under the microscope. The results showed that cassava stomata were kidney-shaped (Figure 1a).

### 3.2 Rhodamine 6G can stain cassava stomata

Previous studies have shown that rhodamine 6G can be used for stomatal staining to measure SA in *Arabidopsis thaliana* [30]. In order to study whether rhodamine 6G could stain cassava stomata, 1-month old cassava leaves were pre-incubated in MES/KOH buffer (5 mM KCl, 10 mM MES (2-(*N*-morpholino) ethanesulfonic acid), and 50 μM CaCl_2_, pH 6.15) under light conditions for 2 h and then stained with 1 μM rhodamine 6G (dissolved in water from 1 mM stock solution in water) for 2 min. The results showed that rhodamine 6G could stain cassava stomata in whole leaves (Figure 1b). We further compared SA index between whole leaves and epidermal peels under 50 μM ABA treatment. The results indicated that SA was significantly decreased in both leaves and epidermises after ABA treatment. Meanwhile, there was no significant difference between epidermises and leaves (Figure 1c).

### 3.3 Appropriate formaldehyde concentration for cell fixation

In *Arabidopsis*, formaldehyde was added to the staining solution [30]. It was known that formaldehyde reduced intracellular glutathione content and cellular oxidative stress capacity [34]. Therefore, the concentration of formaldehyde would be of particular importance. To determine the equal concentration of formaldehyde, cassava leaves were stained with different concentrations of formaldehyde (2, 4, 6, and 8%) after ABA treatment. The results demonstrated that exogenous ABA treatment resulted in stomatal closure with all formaldehyde concentrations. Then, high concentration of formaldehyde had an adverse effect on stomatal movement. Finally, the results of 4% formaldehyde fixation were the closest to the results of epidermises (Figure 1d). Therefore, 4% formaldehyde was suitable for staining cassava stomata.

### 3.4 Rhodamine 6G does not affect stomatal movement

**Figure 2:**
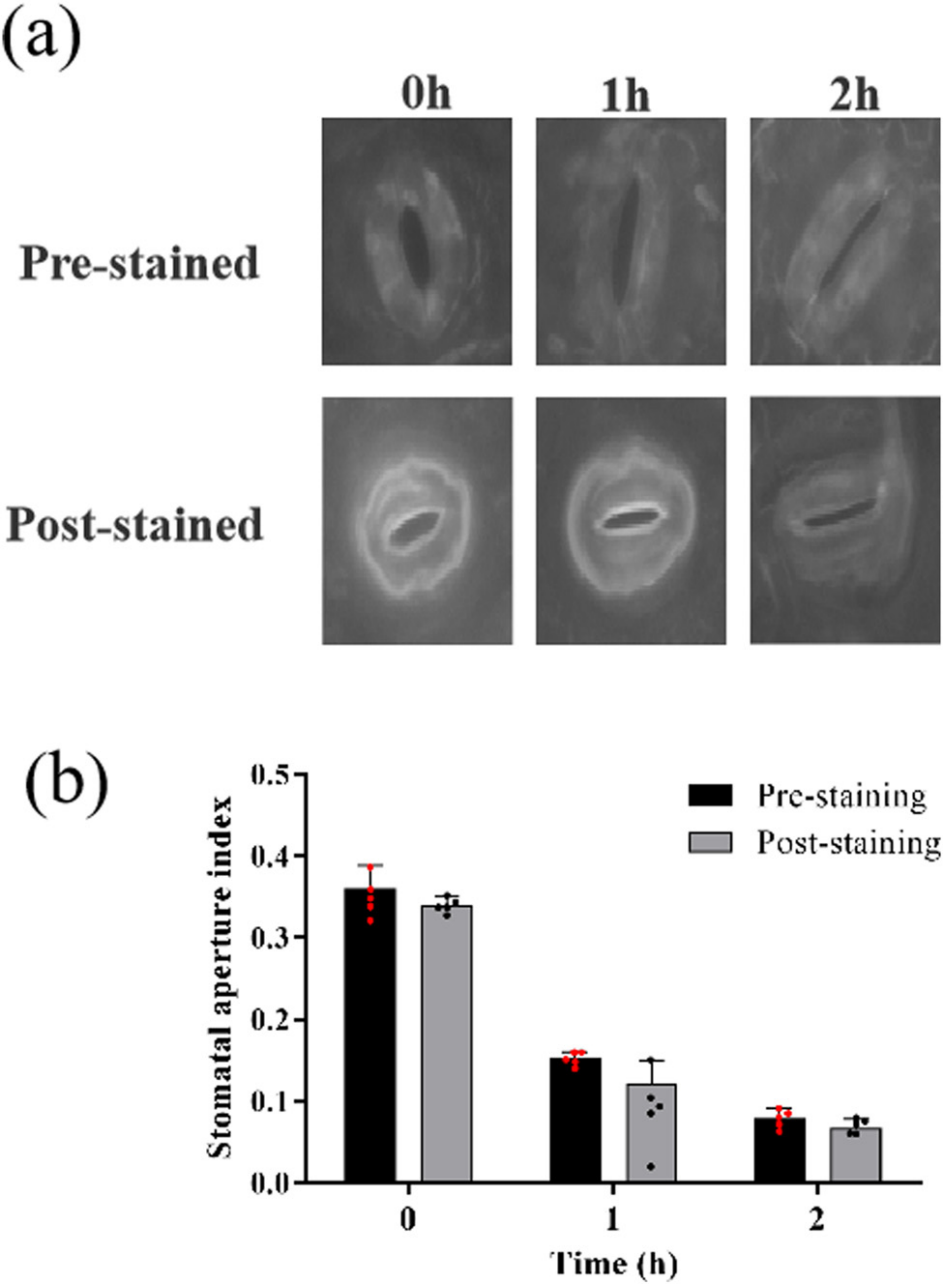
Comparison of pre-stained and post-stained. (a) One-month old cassava leaves were treated with 50 μM ABA for 0, 1, and 2 h after pre-incubated in MES/KOH buffer under light conditions for 2 h and then stained with 1 μM rhodamine 6G for 2 min. (b) The SAs were compared between pre-stained and post-stained after 50 μM ABA treatment.

Two experiments in response to ABA and light, respectively, were designed to detect whether rhodamine 6G could affect stomatal movement during staining. Part of the leaves were stained with rhodamine 6G and then incubated in MES/KOH buffer containing 50 μM ABA (pre-staining). The others were incubated with 50 μM ABA-containing MES/KOH buffer and then stained with rhodamine (post-stained). It was observed that rhodamine 6G staining does not affect ABA-induced stomatal closure (Figure 2a). There was no significant difference in the SA index between pre-staining and post-staining (Figure 2b).

**Figure 3:**
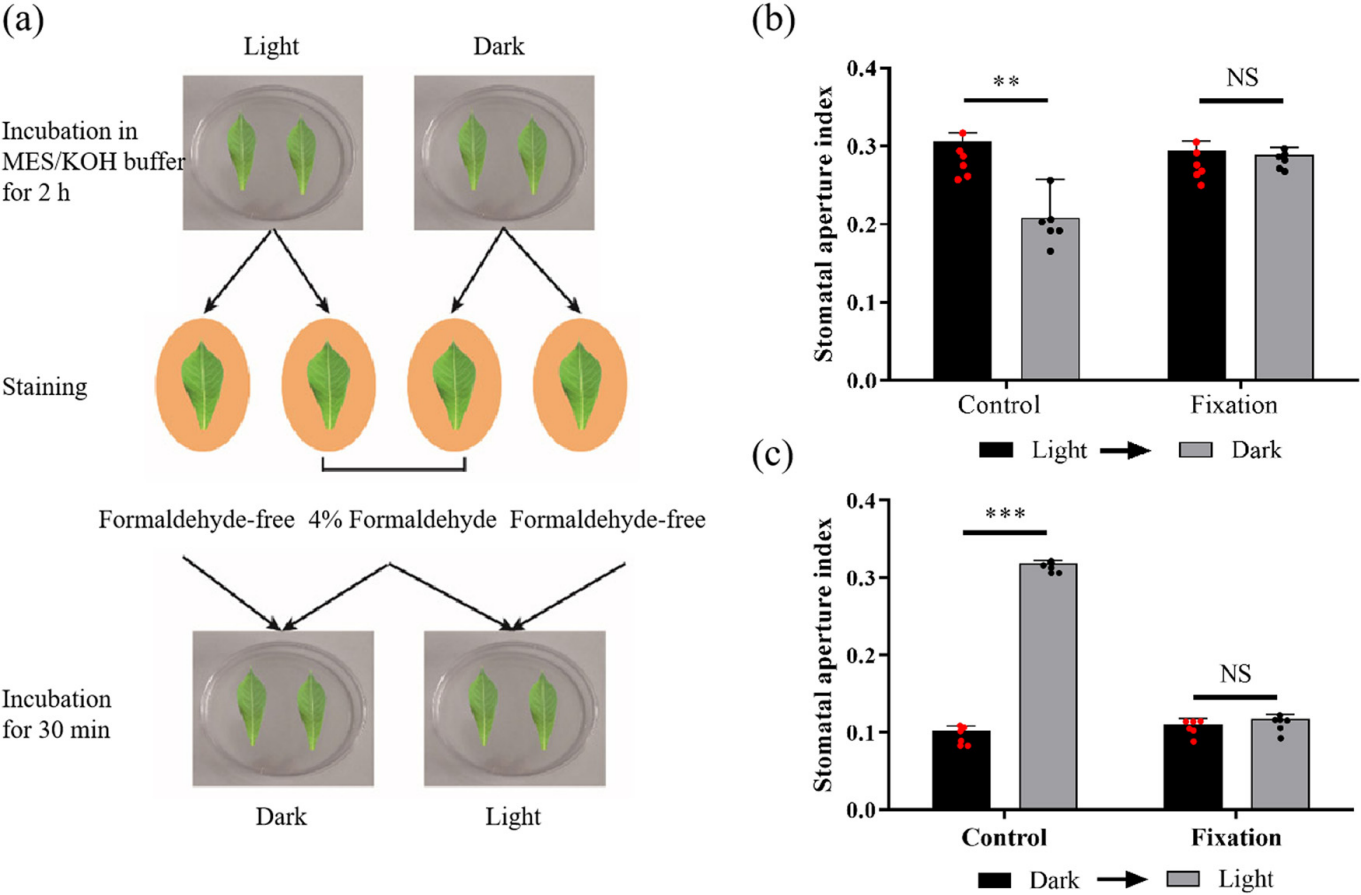
Analysis of stomatal movement under light or dark treatment after rhodamine 6G staining. (a) Flowchart of stomatal movement experiments. Cassava leaves were stained with 4% formaldehyde and formaldehyde-free solution, respectively, after incubating in MES/KOH buffer for 2 h. Then, the leaves incubated under light (dark) were observed after incubating for 30 min under dark (light) condition. (b) First, the leaves incubated in light were analyzed. Second, the leaves were again analyzed after transferred into darkness. (c) First, the leaves incubated in dark were analyzed. Second, the leaves were again analyzed after transferred into light. The leaves stained with formaldehyde-free solution were selected as control. Twelve leaves at a comparable developmental stage were used for analyses. Two asterisks indicate a very significant difference (**, 0.001 < *p* ≤ 0.01), while three asterisks depict extremely significant difference between the results of two subsequent measurements (***, *p* ≤ 0.001).

It was known that light could stimuli open stomata [35]. The leaves were incubated in MES/KOH buffer for 2 h under light and dark conditions, respectively. Then, the leaves were stained by rhodamine 6G with or without 4% formaldehyde. At last, the leaves were incubated in MES/KOH buffer for 30 min under dark and light condition, respectively (Figure 3a). The results showed that the SA index of stomata stained without formaldehyde fixation were decreased obviously after dark treatment, and there was no significant difference between before and after dark treatment in stomata pre-stained by rhodamine 6G with 4% formaldehyde (Figure 3b). Similarly, the stomata pre-incubated without formaldehyde were opened after light treatment, but the stomata pre-incubated with formaldehyde were not opened after light treatment (Figure 3c). These results suggested that rhodamine 6G does not affect stomatal movement, and 4% formaldehyde could almost completely inhibit stomatal movement.

### 3.5 Application of method for measuring the stomata

The method of measuring the stomata could provide technical support for the study of the SA size, SD, and the pattern of stomatal distribution in leaves. We designed two experiments to show the application of method for measuring the stomata.

**Figure 4:**
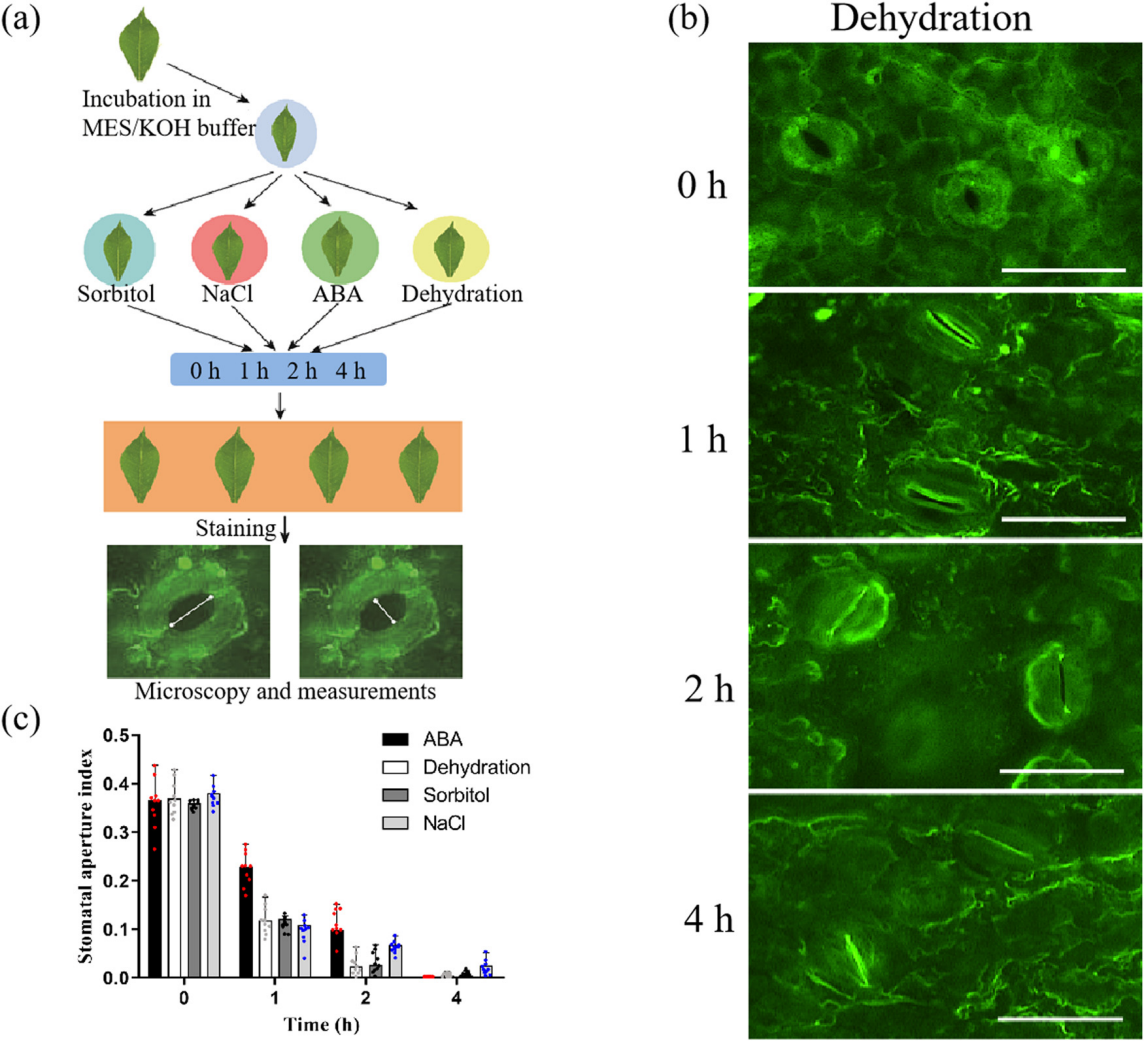
Stomatal movement in response to abiotic stress and ABA treatment. (a) Flowchart of stomatal movement in response to abiotic stress and ABA treatment. Cassava leaves were treated with 50 μM ABA, 150 mM NaCl, 300 mM sorbitol, dehydration, and photos of the leaves were taken after staining by LEICA DM5000 B microscope. Imaging was performed at 0, 1, 2, and 4 h. White lanes were the width and the length of stomata. (b) Cassava leaves were observed under dehydration treatment. Bar = 25 μm. (c) SA in cassava leaves were measured after treated with ABA, dehydration, sorbitol, and NaCl for 0, 1, 2, and 4 h.

**Figure 5:**
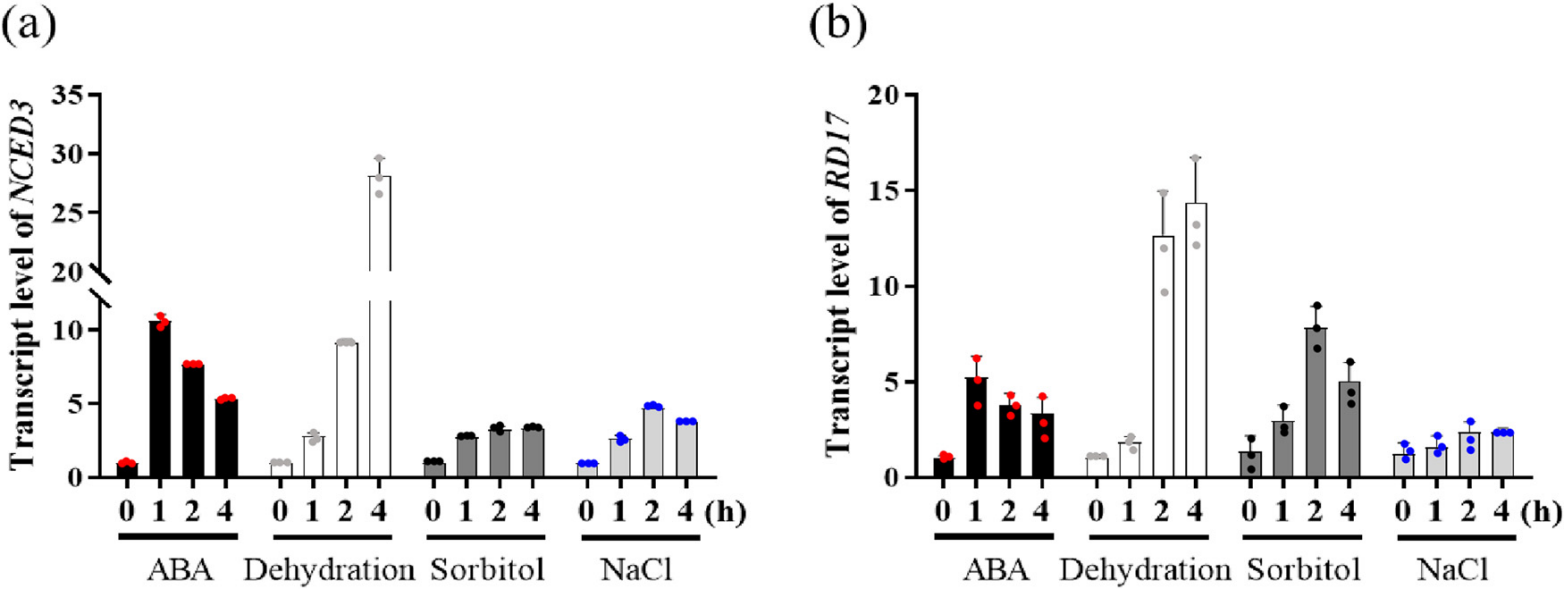
Application model for rhodamine 6G staining. (a) qRT-PCR was used to detect the relative expression of MeNCED3 in different treatments. (b) qRT-PCR was used to detect the relative expression level of MeRD17 in different treatments. Untreated leaves were used as control group. Data were mean (±SD) of three biological replicates.

Cassava leaves were incubated in MES/KOH buffer for 2 h, and then treated with 300 mM sorbitol, 150 mM NaCl, 50 μM ABA, and dehydration. The leaves were detected through microscopy and measured through ImageJ software after staining at 0, 1, 2, and 4 h (Figure 4a). To confirm whether the treatments were effective or ineffective, the expressions of *MeNCED3* and *MeRD17*, which were known to be induced by abiotic stress and ABA, were analyzed by qRT-PCR. The results of microscopy under dehydration treatment showed that stomata gradually closed with the prolongation of the treatment time (Figure 4b). The results of the SA suggested that the SA of cassava leaves decreased significantly after sorbitol, NaCl, ABA, and dehydration treatment (Figure 4c). In conclusion, the method for measuring the stomata could be used to compare the SA between wild type and mutants or different varieties to study how cassava sensed stress signals and adapted to adverse environments through stomata. The transcripts of *MeNCED3* and *MeRD17* were increased after all treatments (Figure 5a and b).

**Figure 6:**
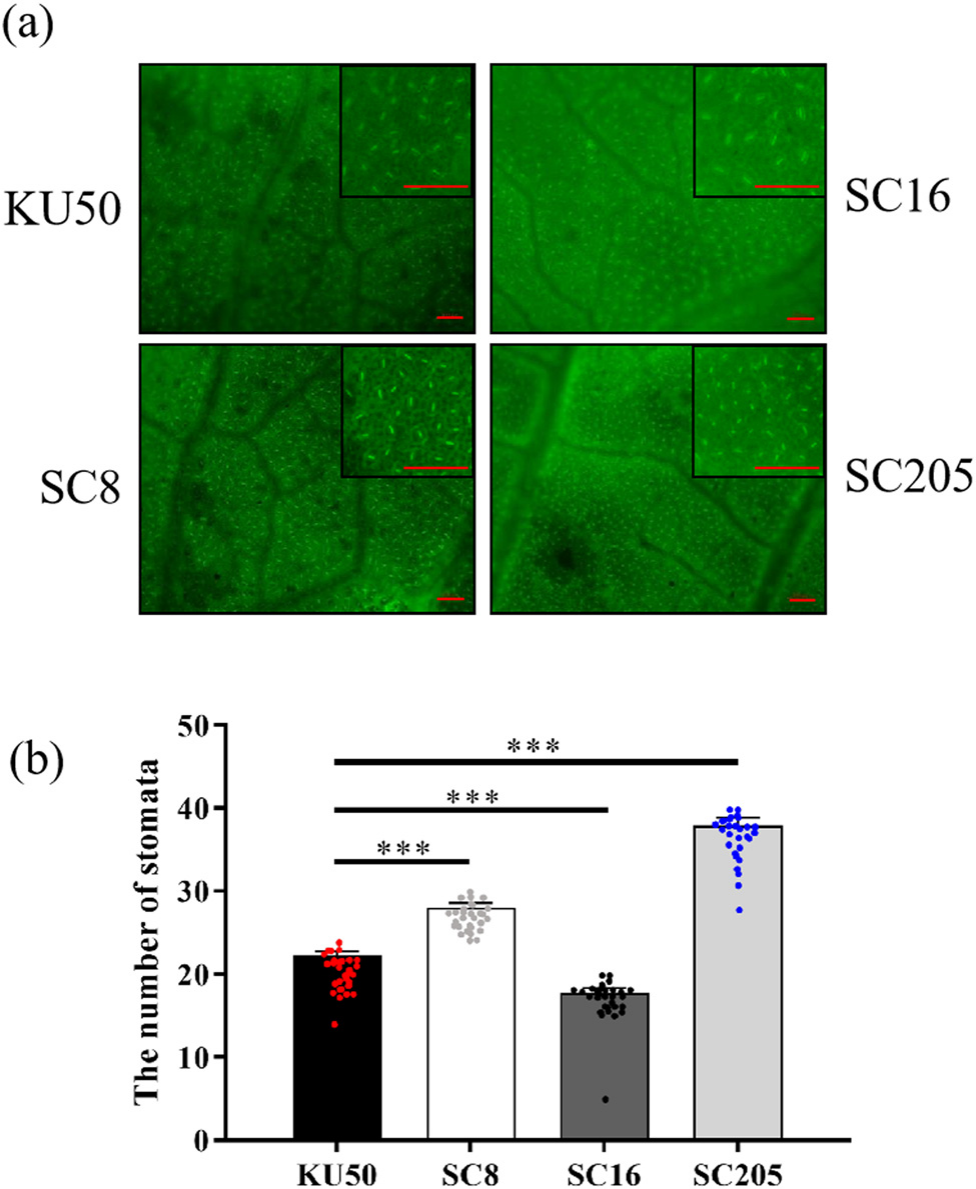
SD of four varieties of cassava. (a) The stomata of four cassava varieties KU50, SC16, SC8, and SC205. Bar = 100 μm. (b) The SD of four varieties was analyzed. Three asterisks depict extremely significant difference between the results of two subsequent measurements (***, *p* ≤ 0.001).

Four cassava varieties KU50, SC16, SC8, and SC205 were selected to count their SD, and it was found that there were differences in SD among four cassava varieties (Figure 6a). SC205 had the highest SD, followed by SC8, KU50, and SC16 (Figure 6b). There were significant differences in SD of different varieties. In summary, the method could also be used to study the mechanism for stomatal development.

## 4 Discussion

Cassava is an important starchy root crop grown globally in tropical and subtropical regions [4]. However, complex environmental changes such as climate drought and soil salinization threaten plant growth and development, reduce crop yield, and seriously affect production and livelihoods [36]. Cassava is one of the most drought-tolerant crops; however, the underlying mechanism for its ability to survive and produce under drought remains obscure [14]. As we all know, transpiration is one of the most basic life activities of plants [37]. When the climate is dry, crop transpiration is aggravated, leading to imbalance of water balance of crop plants and leaf wilting. On the surface of the plant epidermis, a kidney-like organ called stomata plays an important role in plant health under drought conditions. These stomata are like a hole that opens and closes during transpiration. Under drought conditions, lower stomatal transpiration allows plants to escape drought when photosynthetic rates are balanced [38]. In Arabidopsis, guard cell production of γ-aminobutyric acid is necessary to reduce stomatal opening and transpiration water loss. Through the negative regulation of local anion transporters on the tonoplast of stomatal guard cells, it can improve water use efficiency and drought tolerance [39]. Similarly, with the increase of ABA concentration, stomatal conductance of angiosperms rapidly decreases due to drought stress. Ferns close stomata during dehydration to optimize water use efficiency [40]. SS and SD showed opposite effects in response to abiotic stress in rice [41]. In addition, controlling flower stomatal regulation, SS, and SD can be used as a viable strategy to improve the yield of different crops [42]. As can be seen, studies on the stomatal movement of cassava leaves have not been reported.

Currently, there was a stomatal staining method in *Arabidopsis*, but no one has published it yet in cassava. The traditional method of peeling epidermis can be used for stomatal observation. However, peeling epidermis is not only difficult and time-consuming, but also needed to be performed in real time. It can be completed in short time with only limited number of samples, and the consistency of the results cannot be guaranteed for a large number of samples [43]. In this article, rhodamine 6G was applied to cassava stomatal staining for the first time, and the buffer and steps of staining were optimized. The method could complete the fixation and staining of stomata in a fast, simple, and highly efficient manner using distinct microscopy techniques for later detection and statistical analysis. This method could be used to study stomatal development and stress response through two directions of the SA and SD.

The stomata are formed by two guard cells surrounding the stomata, which are surrounded by subsidiary cells that are morphologically distinct from adjacent epidermal cells in many species [17]. Together these are called stomatal complexes. Stomata have two key roles: controlling transpiration (providing nutrients and regulating leaf temperature) and controlling CO_2_ entry into the leaf [44]. Stomatal closure due to insufficient water is a major limitation for photosynthesis and is critical for plant growth and temperature regulation under drought conditions. This study reveals that cassava stomata are kidney-shaped (Figure 1a). Kidney-shaped guard cells first appeared about 400 million years ago and are found in most dicotyledons and some monocotyledons [45]. Dumbbell-shaped guard cells are commonly found in grasses (including most major crops) and other monocotyledons, ferns, and gymnosperms [17]. Stomata with dumbbell-shaped guard cells open and close much faster than stomata with kidney-shaped guard cells, which is due to the greater guard cell membrane surface area to volume ratio [46]. In addition, subsidiary cell type of kidney-shaped guard cells varies between species, whether it is simple patterning for dumbbell-shaped guard cells [17]. Heterogeneity in spatial patterning of kidney-shaped guard cells’ subsidiary cells impacts magnitude and speed of response. The largest difference between dumbbell-shaped and kidney-shaped guard cells is the distribution of cell wall thickness which causes different physiological mechanisms of stomatal movement [45]. So, the cell walls of cassava’s guard cells can be into inner thick wall and outer thin wall.

Formaldehyde was used as a fixative during rhodamine 6G stomata staining in *Arabidopsis* [30]. However, only one concentration was shown in previous study. Considering that formaldehyde is used to cross-link with nucleic acids and proteins, high concentrations of formaldehyde lead to excessive cross-linking and may affect the authenticity of the SA [34]. Thus, the optimal concentration of formaldehyde was needed to analyze. Interestingly, our results show that 6 and 8% formaldehyde seriously affect the results and 2% formaldehyde cannot completely fix stomata. Moreover, we found that staining of rhodamine 6G does not affect stomatal movement (Figures 2 and 3). Previous studies have shown that dehydration, NaCl, and ABA treatment lead to stomatal closure [24,27,36]. Plant cells have developed fine-tuned regulatory mechanisms to orchestrate stomatal movement under drought stress and salt stress. Both drought stress and salt stress can trigger the rapid production of ROS in the apoplast, which is an early hallmark of stomatal movement [47]. ABA is perceived by PYR1/PYL/RCAR family of ABA receptors, then ABA-bound PYLs interact with clade A type 2c phosphatases releasing their inhibition of targets and activating protein kinases, including SnRK2.2/2.3/2.6 (OPEN STOMATA1, OST1) and GUARD CELL HYDROGEN PEROXIDE-RESISTANT1 (GHR1), which inhibit the inward rectifier potassium channel KAT1, resulting in stomatal closure [27]. However, studies on stomatal movement in cassava in response to abiotic stress and ABA are rarely reported. The results show that the stomata are gradually closed under drought, salinity, and ABA treatments, suggesting that stomata play an important role in cassava resistance (Figure 4). Cassava stomatal staining provides technical support for the study of stomatal responses to abiotic stress.

Stomatal conductance is regulated primarily by the SA and SD [17,48]. There is a negative relationship between SD and size. Increased SD often decreases SS, and smaller stomata exhibit faster responses to signaling cues [49]. Furthermore, high SD can have a detrimental effect on stomatal functioning [50]. In dicots, protodermal cell acquires the meristemoid mother cell identity and divides asymmetrically to generate a stomatal lineage ground cell and meristemoid [51]. The meristemoid differentiates into guard mother cell, which divides symmetrically to generate the paired guard cells. In each differentiation state, master basic helix-loop-helix (bHLH) transcription factors, SPCHLESS (SPCH), MUTE, and FAMA govern transition of cell state together with partner bHLH proteins SCREAM1 (SCRM1) and SCRM2 in *Arabidopsis* [51,52]. Our results suggest there are significant differences in SD of different varieties (Figure 5). Many of the genes involved in the developmental pathway of stomata are well established, and genetic modification approaches have successfully demonstrated that reduction of the SD through manipulation can improve water use efficiency at least in C3 plants without impacting yield as well as reducing entry of bacteria and other pathogens [17]. Cassava stomatal staining offers a method for analyzing stomatal development.

**Figure 7:**
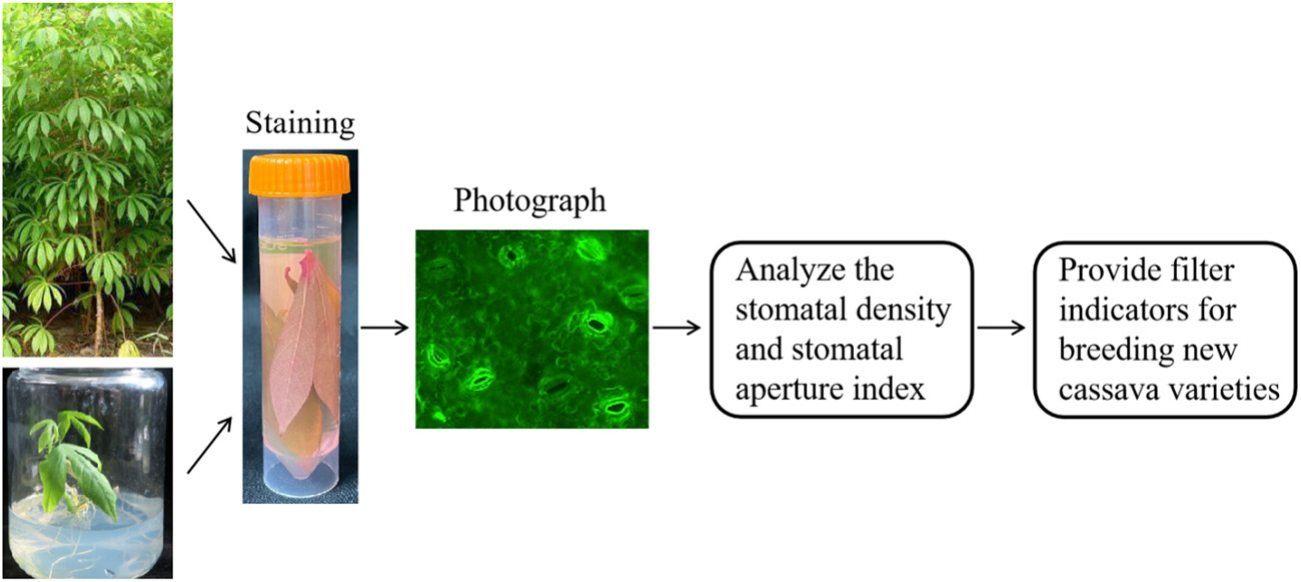
Application model for rhodamine 6G staining.

In conclusion, we explored and optimized a method for staining cassava stomata. We found that rhodamine 6G could stain cassava stomata, and 4% formaldehyde could effectively fix stomata. We found that cassava stomata gradually closed during drought, salt, and osmotic stress treatments. In addition, there were significant differences in SD of different cassava varieties. Moreover, we explored two applications for this method. (1) First, this method could be used to study the mechanism of stomatal movement in response to abiotic stress and ABA. (2) Second, this method could be used to analyze the mechanism of resistance differences among varieties depending on the SD. Compared with the traditional method of observing stomata by peeling the epidermis, our method is more convenient, fast, and practical, especially dealing with a large number of samples. Cassava leaves can be quickly fixed and stained by the pre-configured staining solution. Then, photos of stained leaves can be taken and measured the SD and SA (Figure 7). The method provides new filter indicators for breeding new cassava varieties.

The leaves were sampled into staining solution from cassava grown in soil or medium. Stomata were fixed and stained by rhodamine 6G including 4% formaldehyde. The photographs were taken by microscope at any time. Then, the SA and SD were measured and analyzed.

## Supplementary Material

Supplementary Table
