# Supplementary material for: An optimization method for measuring the stomata in cassava (Manihot esculenta Crantz) under multiple abiotic stresses
Source: Open Life Sci. 2024 Nov 11;19(1):20220993. doi: 10.1515/biol-2022-0993 (PMC11554558; doi:10.1515/biol-2022-0993)
Supplement: Supplementary Table [file biol-2022-0993-sm.pdf]

Supplementary material

Table S1: Primers used in this study

Primer	Sequence (5′–3′)
Ubiquitin-F	GCCTCCCAAGGTAGCTTTCA
Ubiquitin-R	GGTTAATGCAGGGCTCCACT
NCED3-F	TGGGATGGTTCATGCTGTCC
NCED3-R	TATCCCAGAGTGGCCATGGA
RD17-F	CCCAGTTGTTGTTGAGGGGT
RD17-R	GTGACGATCACCTCCCCTTG
